# Nano-Based 3D Printed Scaffold for Bone Tissue Engineering

**DOI:** 10.3390/bioengineering13050569

**Published:** 2026-05-18

**Authors:** Xiaoting Shi, Keda Liu, Weiqi Li, Ruobing Zhao, Wei Wang

**Affiliations:** Liaoning Provincial Key Laboratory of Oral Diseases, School and Hospital of Stomatology, China Medical University, Shenyang 110001, China

**Keywords:** 3D printing, tissue engineering, nano, bone defect

## Abstract

3D bioprinting technology has made great strides in the field of bone tissue engineering. It has been able to create personalized biological structures on a macroscopic scale. In terms of microstructure bionics, 3D printing technology has also made some progress in recent years. The use of nanotechnology and drug delivery technology has provided a microenvironment that is more compatible with cell growth. Finally, it is possible to bridge the gap between engineered organizational structures and natural tissues. In this work, we summarize the widely used 3D bioprinting methods and the preparation of bioinks. Next, the classification of bone tissue engineering scaffold materials and nanomaterials for loading is briefly introduced. Then the technical shortcomings of current nanotechnology-based 3D bioprinting are described, along with the corresponding improvements. Finally, we summarize the prospects of nano-based 3D bioprinting technology in bone tissue engineering.

## 1. Introduction

Large segmental bone defects caused by various reasons are one of the thorniest problems often encountered in orthopedics. The commonly used method is bone grafting. Although, there are many studies on xenograft tissue engineering scaffolds, such as Keil bone, Bio-Oss bone. The problems faced by xenograft bone have not been completely solved. For example, the bone graft material needs to match the shape of defect and transitional properties of cortical cancellous bone [1]. 3D printing technology allows the design and preparation of a scaffold with induced osteogenic activities that matches the shape and anatomical structure of the target location of the bone defect [2,3]. The specific design ideas include the following aspects: (1) Macro-structure bionic: by designing the bionic scaffold consistent with the shape and anatomical structure of the target bone defect area, 3D printing can make the graft material rely on the dense cortical-like structure to ensure sufficient compressive strength, promoting the fusion of cancellous bone. (2) Micro-structure bionic: by changing the pore size parameters inside the 3D porous scaffolds, different material elastic moduli can be obtained to match the elastic moduli of cancellous bone in the fractured end region. (3) Nanotechnology and nanoparticles: by nano-modifying the graft material or loading it with growth factors and nano-drugs, the osteogenic ability of graft materials is promoted [4,5,6].

**Figure 1:**
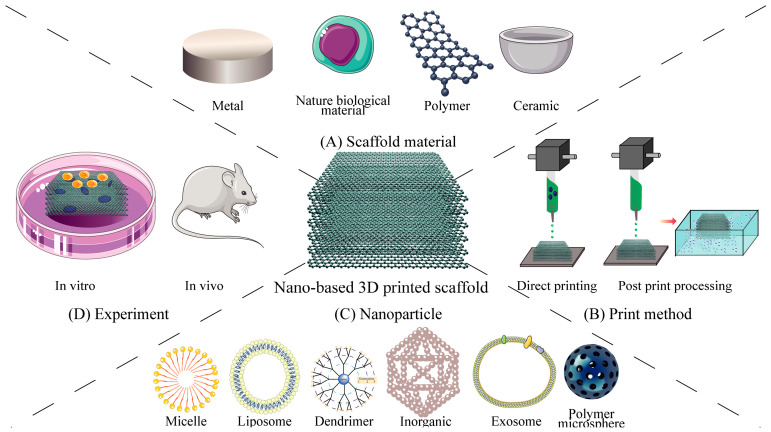
Schematic diagram of 3D printed loaded nanoparticle scaffolds: (**A**) Scaffold material; (**B**) nanoparticle loading mode; (**C**) nanoparticle composition; (**D**) experiment.

The following sections mainly review the classification of 3D printing technology applied to the field of bone defects, the composition of 3D printing scaffold materials, preparation of bioinks for 3D printed scaffolds and the nanoparticles loaded by 3D printed scaffolds. Schematic diagram of nano-based 3D printed scaffolds is shown in Figure 1.

## 2. 3D Printing Technology

3D printing is a new technology that emerged in the late 1980s. Its principle is to form 3D entities by adding materials layer by layer. 3D printing is based on the same principles and printing methods as ordinary inkjet printers. The major difference is that the nozzles of 3D printers can move not only in the plane, but also move vertically. For the 3D printing of tissues/organs, the basic steps are firstly, obtaining the tomographic imaging data of the patient’s diseased/defective tissue, using computer-aided design to build an image model and scanning according to various parameters. The virtual “slices” are then fed into a 3D printer. Basing on these ultra-thin “slices”, suitable biomaterials are printed layer by layer and superimposed to form a three-dimensional scaffold that conforms to the tissue. The greatest advantage of 3D printing is that it can accurately and quickly realize the integrated construction of complex macroscopic shapes and internal microstructures of scaffolds and can achieve personalized production for specific tissues and organs, which cannot be reached by traditional biomaterial manufacturing [7,8,9,10].

### 2.1. Stereolithography

**Figure 2:**
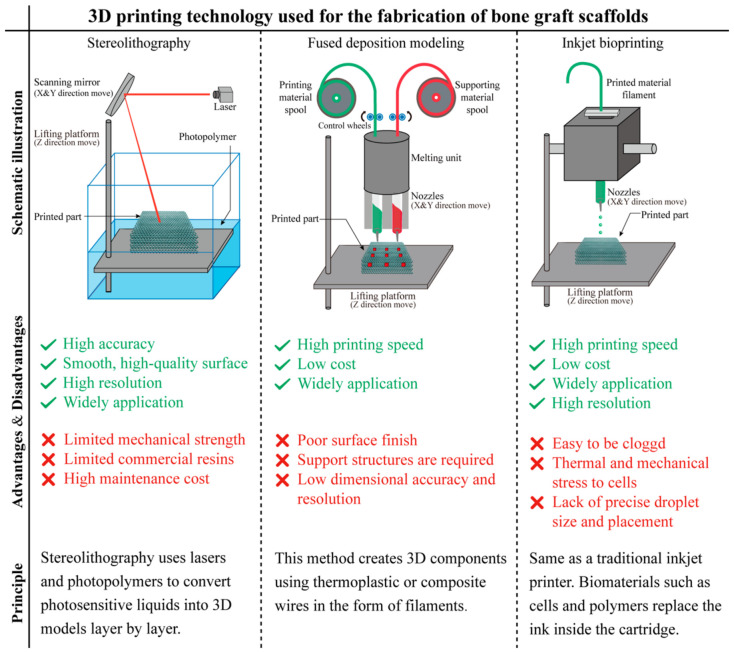
Schematic illustration of Stereolithography, Fused deposition modeling and Inkjet bioprinting [16,17,18,19,20,21].

SLA is the abbreviation of stereolithography. It is generally regarded as the oldest 3D printing technology. The foundational principles of this technology can be traced back to the early 1980s. In 1980, Hideo Kodama filed the first patent application (JP S55-48210) for a rapid prototyping system using photopolymers. He subsequently published the fundamental principles of stereolithography in 1981 [11]. Around the same time, A. J. Herbert also described similar concepts of solid object generation by writing on a photopolymer surface in 1982 [12]. Later, in 1986, Chuck Hull filed a patent for the “Apparatus for Production of Three-Dimensional Objects by Stereolithography” (US4575330A), which led to the commercialization of the technology. In 1988, the 3D printing industry giant 3D Systems produced the world’s first SLA 3D printer, SLA250, based on the principles of SLA molding technology, and commercialized it [13]. Since then, models based on SLA technology have appeared one after another. Of all 3D printing technologies, SLA is the gold standard: The earliest 3D printing technology with high maturity; good surface quality and high precision (about 10 μm); diverse printing materials and various functional materials, which can be selected according to one’s needs; the prototype is directly generated from CAD data, with fast processing speed, and a relatively short production cycle. However, post-processing steps such as solvent cleaning to remove uncured resin, support removal, and UV post-curing are typically required to achieve final mechanical properties. While traditional laser-based SLA offers a balance of speed and precision, it is worth noting that high-resolution fabrication typically comes at the cost of build speed. However, recent advancements such as Continuous Liquid Interface Production (CLIP) have significantly improved volumetric build rates. But the shortcomings of SLA technology are also obvious: The cost of the system and the maintenance is high; the use of environment requires controlled lighting to prevent premature curing and proper ventilation for resin handling; furthermore, achieving high-quality prints necessitates the precise optimization of multiple parameters, such as light exposure conditions, material properties, and optical characteristics, which requires a certain level of expertise [14]. SLA uses a laser to focus on the surface of the light-cured material, so that it solidifies sequentially from point to line and line to surface, and repeated and superimposed to form a three-dimensional solid [15]. The schematic diagram and characters of SLA is shown in Figure 2.

Researchers used SLA technology to synthesize a 3D printed scaffold with a biphasic bionic structure for osteo-cartilage regeneration. The scaffold contains nano-hydroxyapatite and transforming growth factor β1 nanoparticles, which are respectively distributed in the lower and upper layers of the scaffold. This scaffold successfully promoted osteogenesis and cartilage differentiation of human bone marrow mesenchymal stem cells and enhanced gene expression associated with osteogenesis and cartilage generation. This 3D printed osteochondral scaffold with a bionic biphasic structure is an excellent candidate for osteochondral repair and regeneration [22].

It is important to note that stereolithography encompasses a broader range of technologies beyond laser-scanning systems. Digital Light Processing (DLP), for example, utilizes a digital micromirror device to project a complete layer image simultaneously, offering faster print speeds for certain geometries. Furthermore, Two-Photon Polymerization (2PP) represents the cutting edge of high-resolution stereolithography, capable of fabricating sub-micrometer features with complex 3D architectures, such as the Nanoscribe systems [23]. These advancements highlight the diversity and potential of light-based 3D printing for nano-scale fabrication.

### 2.2. Fused Deposition Modeling

Fused deposition modeling, FDM, also known as fused deposition. This method works by heating and melting a filamentous hot-melt material and extruding it through a fine nozzle [24]. Extruded from the nozzle, the thermoplastic material is heated above its melting temperature. During deposition, the previously deposited layer is partially remelted, enabling interlayer thermal fusion and ensuring bonding between layers [25]. After the deposition of one layer is completed, the workbench reduces the thickness of the layer by predetermined increments, and then continues to melt-blown deposition until the entire solid modeling is completed [26]. The fused deposition rapid prototyping process needs to make supports during prototyping. In order to save material costs and improve deposition efficiency, the new FDM equipment adopts dual nozzles. One nozzle is used to deposit the model material, and one nozzle is used to deposit the supporting material. The support material can be either a water-soluble material or a hot-melt material below the melting point of the model material to facilitate later removal [27,28,29]. Schematic diagram and characters of FDM is shown in Figure 2.

The PCL/nHA/CNW (PHC) nanocomposite scaffold is a 3D printed scaffold manufacture by FDM. In this study, nanocomposite filaments for FDM printing were prepared using a variety of nanomaterials such as nanohydroxyapatite (nHA), chitin-nano-whiskers (CNW) and polycaprolactone (PCL). The results of materials science experiments showed that the CNW content improved the mechanical properties of the scaffolds and promoted cell proliferation and adhesion. nHA content greatly improved the mechanical properties of the scaffolding. nHA and CNW nanofillers increased the biodegradation rate of PCL [30]. The latest research used FDM technology to manufacture PLA/β-TCP scaffolds. The researchers then took advantage of the surface adhesion ability of polydopamine to dope nano-Ta on the scaffold surface to improve its properties. The composite material had good physical properties. In vitro studies showed that the scaffold significantly promoted cell proliferation, migration, and osteogenic properties [31]. FDM is a fast and efficient 3D printing technique. Researchers often mix nanomaterials in fusible filaments to prepare composite scaffold. It is worth noting whether temperature will change the physical and chemical properties of the material.

### 2.3. Inkjet Bioprinting

Inkjet printers use instantaneous heating or ultrasonic pressure at the nozzle to break down liquid bioink into small droplets and print them on a specific material substrate [32]. Inkjet-based bioprinting draws upon the principles of conventional inkjet printing, offering potential advantages in terms of resolution and speed [33]. However, directly applying the characteristics of standard inkjet printers to bioprinters requires caution, as bioprinters necessitate stringent requirements such as sterile environments, precise temperature control, and considerations for maintaining cell viability, which can increase system complexity and cost. Additionally, since inkjet printers use nozzles for printing, bioink with too high cell density cannot be used, otherwise the nozzle is easily clogged, and make it difficult to print high-density tissue. Studies have shown that the viscosity of bioinks needs to be less than 10 mPa·s. The cell density needs to be less than 106 cells/mL [34,35]. In addition, thermal inkjet printers use instantaneous heating to obtain droplets, which print with low directionality and uneven size. Acoustic inkjet printers use high frequencies from 15 to 25 kHz that may induce cell membrane damage and lysis [32]. The schematic diagram and characters of Inkjet bioprinting is shown in Figure 2.

Recent studies have been conducted to develop cytotic artificial meniscus using inkjet printing technology. Before printing, primary mesenchymal stem cells (MSCs) were isolated from the bone marrow and mixed into collagen-based bio-inks. The researchers used inkjet printing to achieve collagen meniscus filled with MSCs. The implant material is well biocompatible and patient specific. This can help optimize implants designed to replace damaged meniscus [36]. Studies have reported that silver nanoparticles (nAg) were immobilized in lactose-modified chitosan (chitlac) to prepare bacteriostatic coatings. This functional chitlac-nAg coating was subsequently deposited in a commercially available thermoset polymer matrix using inkjet printing technology to repair skull defects. Inkjet printing technology enables controllable, fast and flexible functionalized coating patterns for individual customization [37].

### 2.4. Laser-Assisted Bioprinting

**Figure 3:**
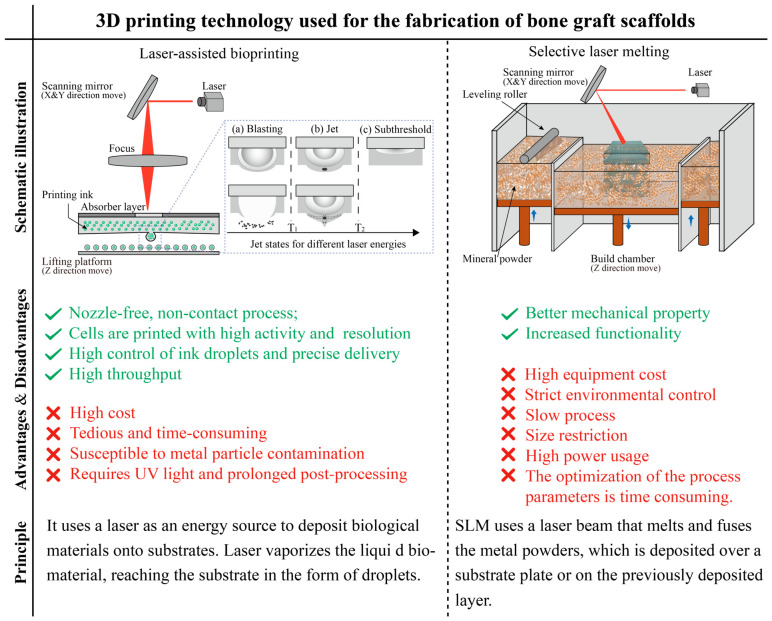
Schematic illustration of laser-assisted bioprinting and selective laser melting [42,43,44,45].

Laser-assisted bioprinting, LAB, is a printing technology based on the principles of laser-induced forward transfer (LIFT). Originally developed as a technique for thin-film transfer and 2D micropatterning, LIFT has evolved into a powerful tool for additive manufacturing. By repeatedly depositing materials in a layer-by-layer fashion, it enables the construction of 3D structures with high resolution and cell viability. LIFT is a direct-writing technology that was originally developed to transfer metals, but later improved to successfully transfer peptides, DNA, cells and other biological materials [38]. Lift devices for bioprinting are generally composed of a laser source, a target plate and a receiving substrate [39]. The target plate typically consists of a transparent substrate (typically a treated transparent quartz slide), an absorbent layer (e.g., gold or titanium), and a bio-ink coating. The process is: laser energy deposition heating absorption layer—heat transfer to the top of the absorbent layer of bio-inking film—bio-ink interface deformation between bio-ink and air—to produce jets [40]. The key technologies of LAB include three parts: laser modulation, target plate preparation, and receiving substrate processing, which correspond to laser parameters (wavelength, pulse width, repetition rate, energy, etc.), transparent substrate, absorption layer, and bio-ink properties (viscosity, thickness, surface tension, etc.), receiving substrate composition, etc. Jet generation relies on the extrusion of air bubbles and, according to different energy, the jet will show three states: subthreshold, jet and blast, as shown in Figure 2. The laser pulse fluence must exceed a minimum threshold for droplet ejection to occur. When the laser energy density T < T2, the cavitation bubbles are not sufficient to generate jets, while when the energy density T > T1, the bubbles rupture of the surface, producing submicron droplets. Jets can only be generated when the energy density is at the intermediate value T1 < T < T2 [40]. Increasing the viscosity or thickness of the bio-ink film increases the laser flux threshold. To ensure successful printing, the metal absorber layer must have a high absorption coefficient and be slightly thicker than the metal’s skin depth. The receiving substrate is usually coated with a hydrogel to keep the biomaterial active after printing. Increasing the viscosity of the bio-ink by sodium alginate can improve cell viability [41]. Schematic diagram and characters of LAB is shown in Figure 3.

Using BioRoot RCS^®^ (Septodont, Saint-Maur-des-Fossés, France) as a mineral additive to collagen-rich inks, the researchers developed cured tricalcium silicate inks as a new matrix to promote bone repair. The study then utilized LAB technology to make this ink serve as a new bone repair promoting matrix. This ink formula exhibits good cytocompatibility and has a positive effect on cell motility without affecting the vascularization of newly formed tissues [46]. LAB is an attractive tool for in situ printing of bone substitutes due to its cellular printing resolution and accuracy. LAB allows design patterns to be printed in a non-contact manner. Unlike extrusion and inkjet bioprinting, LAB eliminates the possibility of nozzle blockages and cells subjected to shear pressure. However, this method is the most expensive and complex 3D printing method, which limits its commercial application [38].

### 2.5. Selective Laser Melting

Selective laser melting, SLM, is a main technical approach in the additive manufacturing of metal materials. The technology selects laser as the energy source, scanning the metal powder bed layer by layer according to the path planned in the 3D CAD slice model [47]. The scanned metal powder is melted and solidified to achieve the effect of metallurgical bonding, resulting in the design model of the metal part. SLM technology overcomes the difficulty of manufacturing complex-shaped metal parts by traditional processes. It allows the direct formation of precise metal parts with good mechanical properties [48]. Another advantage is that the surrounding unmelted powder can provide support for the printed structure, potentially reducing the need for dedicated support structures compared to other technologies. However, SLM systems typically require high equipment costs due to the need for high-power lasers and strict environmental controls, such as inert gas atmospheres, to prevent oxidation. Schematic diagram and characters of SLM is shown in Figure 3.

Researchers have used SLM-titanium to improve bone regeneration. This study then uses anodization (SANs) and alkali-heat treatment (SAH) to produce ordered nanotubes and disordered nano-meshes. The results show that SAH’s three-dimensional nano-mesh is more like the natural extracellular matrix (ECM), which leads to better bone formation than nanotubes [49]. The advantage of 3D printing is that personalized bone implant materials can be prepared. In order to solve the mismatch between solid metal orthopedic implants and the elastic modulus of human bone tissue, researchers prepared metal structures with excellent mechanical properties by using SLM. They compared the effects of different pore shapes and porosities on the compressive yield strength and elastic modulus of the porous structures. This means that future researchers can repair bone defects using bone implant materials with gradient modulus of elasticity [50].

## 3. 3D Printed Nano-Scaffold

The latest 3D printing technology allows us to easily manufacture special materials with complex microstructures, which can open new opportunities for materials development. Due to the specificity these materials exhibit at the micro- and nanoscale they are widely utilized in the development of modern materials with customized and improved properties. The unique structures are designed to exploit their potential capabilities and advances in multidimensional manufacturing to facilitate their application in industry. Nano-scaffold materials can be broadly classified as metals, ceramics, nature materials or polymer composites. This section mainly reviews the composition and properties of nano-scaffold materials. The characteristics of different materials are summarized in Table 1.

### 3.1. Metal Material

In the fields of stomatology and orthopedics, alloys prepared by 3D printing combined with nanotechnology have been widely used. At present, it mainly includes two research directions. (1) To precisely adjust the internal pore structure of the material through additive manufacturing technology according to the site of the bone defect, so that the new alloy material has excellent biomechanics and good osteoinductive properties. (2) Achieving better osseointegration by changing the nanomorphology of the implanted material surface [51].

**Figure 4:**
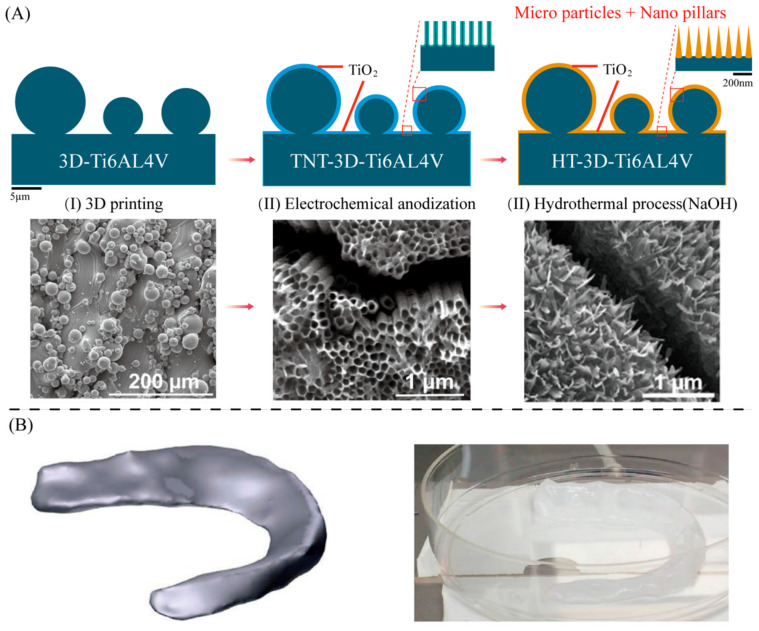
Schematic diagram of 3D printing technology based on metal and natural biological materials. (**A**) The schematic presentation of the concept for the fabrication of Ti materials. First, SLM is used to obtain Ti implants with a spherical surface structure. Subsequently, the nanotube structure was obtained by electrolytic etching surface treatment. Finally, the nanocolumnar structure was obtained by hydrothermal process. Reproduced with permission from Copyright © 2021, American Chemical Society [52]. (**B**) STl model of a human meniscus and custom high-density type I collagen meniscus embedded with mesenchymal stem cells. Copyright © 2019, The British Editorial Society of Bone & Joint Surgery [36].

There is a growing clinical demand for low-cost and more efficient Ti medical implants. Ti alloys (Ti6Al4V) printed using SLM have more structural surfaces. This new material has a unique dual micro-nanomorphology, consisting of micron-level spherical features, fabricated by vertically aligned nanoscale pillar structures. It enhances hydroxyapatite-like mineral deposition in simulated body fluids. Normal human osteoblast-like cells osteoblasts exhibit strong adhesion to nano/microstructures and exhibit greater tendency to mineralize [52,53]. Figure 4A shows the schematic presentation of the concept for the fabrication of Ti materials. Biomimetic multi-structural surfaces are created on SLM-printed Ti6Al4V implants by electrochemical anodizing and hydrothermal (HT) processes. Some researchers have used acid etching to change the surface roughness of 3D printed Polyetheretherketone (PEEK) titanium composites (PTCs), resulting in micro- and nano-scale structures. This approach helps maintain hBM-MSCs viability and triggers the expression of early osteogenic markers [54].

3D printing additive manufacturing technology helps to prepare porous and personalized titanium implants. It is generally accepted that porous scaffolds should have a porosity of >60% or/and a pore size of >300 μm [55]. However, whether there is a correlation between pore size and porosity in accelerating osteointegration requires further investigation. Specific load-bearing conditions should also be considered for printing implant materials within the design recommendations. Post-printing modifications such as acid etching, sandblasting and electrolysis can help to change the surface morphology of the implanted material, which has a positive effect on accelerating osteointegration. Post-print modifications need to be further studied [56].

### 3.2. Nature Biological Material

The biological 3D printer is a technology for printing biological tissues based on computer programming technologies that allow precise control of the distribution, interaction and differentiation of biological materials, cells and other biological factors on the 3D skeleton [57]. By integrating relevant knowledge from multiple disciplines such as medicine, engineering, electronic informatics, and biology, 3D bioprinting technology has been able to print implantable biological products that can completely replace human organs or tissues [58].

Using 3D printing technology to artificially create a functioning organ requires three necessary conditions: cells, scaffolds, and induction. (1) Cells are prerequisite for 3D bioprinting. While ensuring the biological activity of cells, it is necessary to accurately drop the cell-encapsulated droplets from the nozzle of the printer. In addition to ensuring a sterile environment, the density and location of cell distribution are critical for bioprinters to successfully print biological tissues. (2) Scaffold simulates the skeleton of the target organ, and cells are accurately distributed by attaching to the scaffold and finally constitute a three-dimensional biological organ or tissue. The scaffold must be a non-toxic, biodegradable, non-contaminating biological product suitable for cell proliferation. The density of the scaffold and the mechanism of cell arrangement need to be solved urgently. (3) Precise induction conditions are the biggest difficulty in organ printing [59,60]. As we all know, tissues and organs are composed of a variety of functional cells and in dynamic change. It is not biologically meaningful to anchor some cells to a scaffold to form an organ, and mature cells cannot be reprogrammed to acquire new functions. Therefore, the current bio-3D printing is mostly used to construct stem cell scaffolds. The consequent problem is the induction of stem cell differentiation. The problem is complex and wide-ranging, and cells are affected by various factors such as proximity interference, cellular environment, chemical signals, and physical signals [61].

Live cell printing, which has attracted particular interest in the field of tissue engineering scaffolds, aims to more closely reproduce the 3D microenvironment of the target tissue. Studies have reported that the use of shark collagen at the nanoscale will eventually produce a stable ink, encapsulated adipose stem cells (hASC) to prepare 3D printing scaffolds. It promises to be a bioactive bio-ink with potential properties for bone tissue regeneration [62,63]. Figure 4B shows that the researchers developed a cellularized artificial meniscus using 3D bioprinting. A 3D model of the medial meniscus tissue was created based on MRI scans of human volunteers. Primary mesenchymal stem cells (MSCS) were isolated from bone marrow and embedded in collagen-based bioink prior to printing. An artificial meniscus was made based on inkjet printing technology [36].

### 3.3. Ceramic Material

3D printing of ceramic parts includes processes such as configuring ceramic paste, drawing 3D models and slicing, 3D printing molding, and sintering. It can directly generate objects of any shape through additive manufacturing based on computer graphics data, which simplifies the product manufacturing process, shortens the development cycle, improves efficiency and reduces costs. The ceramic parts obtained by 3D printing technology can be obtained after high temperature degreasing and sintering [64,65,66].

**Figure 5:**
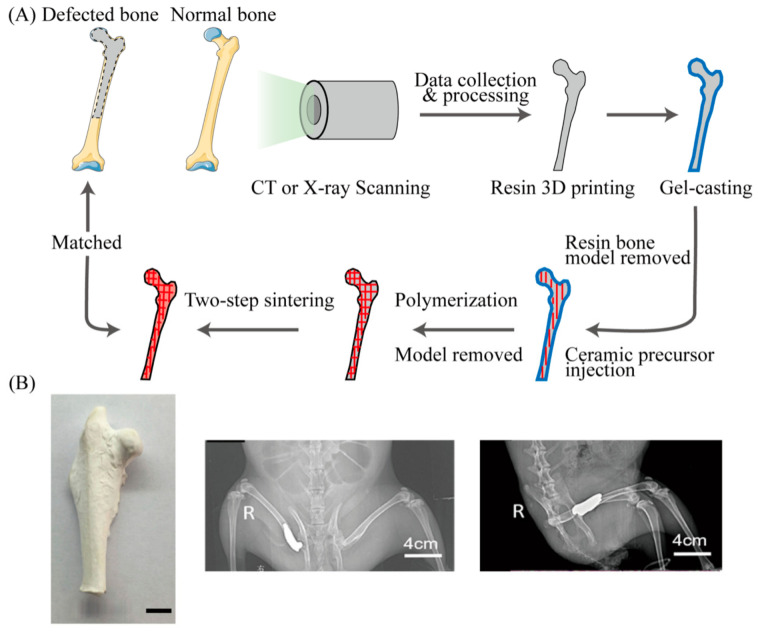
(**A**) Schematic diagram of the manufacturing process of a ceramic hip joint prosthesis combined with 3D printing technology by gel casting method. (**B**) Material samples and X-ray patterns of the ZrO2-ZnO ceramic hip prosthesis after implantation. Reproduced with permission from Copyright © 2019, Dovepress.

Zirconia ceramics are becoming the material of choice for metal-free dental implants, but considerable efforts are required to obtain rough/porous surfaces to enhance osteointegration and avoid the risk of surface delamination and microstructural changes. Researchers used nanoparticle additive to create zirconium oxide implants with integrated layered surface morphology. The dense core of this implant combines seamlessly with a porous surface, having a unique surface with directional layered pore morphology. This work demonstrates the opportunity to 3D print new ceramic implants that can meet both mechanical and biological functional requirements [67]. Two technical problems with 3D printed ceramics (brittle and irregular shrinkage caused by sintering) are the main factors limiting their development. The study combined ceramic 3D printing with ZnO antibacterial nano-modification to obtain implant materials with precise 3D structure and effective antibacterial properties. Both of these technical problems were effectively solved by optimizing the reaction conditions and selective area inversion compensation. At the same time, ZnO modification has no significant effect on the mechanical properties of ZrO and can effectively inhibit bacteria [68]. Figure 5A shows the schematic diagram of the process of 3D printing ceramics.

### 3.4. Polymer Composites

With excellent physical and chemical properties, good biocompatibility and degradability, polymer composites have become the fastest growing printing materials in the field of biological 3D printing [69]. At this stage, 3D printing has extremely high application value in the medical field, mainly used in the fields of orthopedics and dentistry [70]. According to medical needs, staff can use 3D printing technology to print personalized organs, bones, tissues, etc., which can be used as medical models, tissues or implants for medical course teaching, surgical simulation or clinical treatment, with irreplaceable roles and advantages in the medical field [71,72].

**Figure 6:**
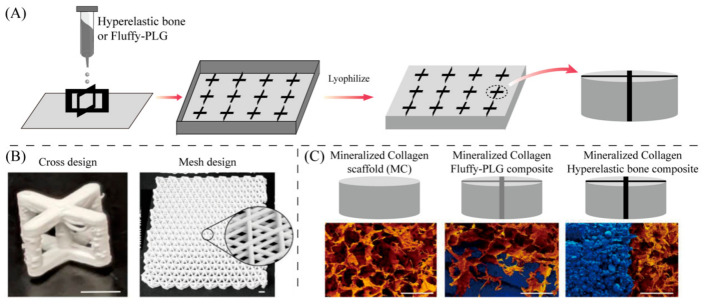
(**A**) 3D-paints were 3D printed in cross designs. Scaffolds were then prepared by immersion in mineralized collagen suspension. (**B**) Images of cross and mesh 3D prints. (**C**) SEM images of scaffold composites. The images are false-colored to demonstrate collagen (orange) and 3D-print (blue). Reproduced with permission from Copyright © 2022, ElSEVIER.

Polycaprolactone (PCL) has been widely used in the construction of 3D printed tissue engineering scaffolds. However, it has inadequate mechanical support, slow degradation rate and lacks bioactivity and cell induction capabilities. Studies have reported the preparation of 3D PCL scaffolds using novel magnesium-doped bioactive glass (Mg-BG) for these problems. The material promotes cell viability and supports the mechanical load of the host trabeculate [73,74]. The researchers incorporated 3D printed polymer or ceramic-based grids into mineralized collagen scaffolds to improve mechanical and biological activity. The mineralized collagen scaffold is reinforced with a 3D printed Fluffy-PLG (ultra-porous polylactide-coethanolide copolymer) or superelastic bone (90 wt% calcium phosphate in PLG) meshes. The grid formed by hyperelastic bone significantly improves the mechanical properties of the composite and causes a significant increase in the secretion of osteoprotegerin as is shown in Figure 6.

**Table 1:** Summary of the types and characteristics of the scaffold printing materials.

Material	Types	Features	
Metal	Stainless steel alloys, titanium and titanium-based alloys, nickel-based alloys, cobalt-chromium alloys, tantalum, aluminum alloys, magnesium, gallium alloys, iron, copper alloys and precious metals, etc.	Good mechanical properties, corrosion resistance and biocompatibility but the price is high, harmful ions may be released and some metal materials are unstable.	[75,76,77,78,79,80,81]
Nature biological	Collagen, chitin, coral, chitosan and its derivatives, etc.	Good biocompatibility, no toxic side effects, abundant resources, low price, insufficient mechanical properties.	[57,82,83]
Ceramic	Hydroxyapatite, demineralized bone matrix and coral, calcium silicates and bioactive glasses, etc.	Inherent biocompatibility and bone bioactivity.	[84,85]
Polymer	Acrylonitrile butadiene styrene, Polycarbonate, Polyether ether ketone, Polyethylene terephthalate glycol, Polylactic acid, Polyamide 12 (Nylon), Acrylic-based, Epoxy-based, Methacrylic Acid, etc.	Natural polymers are biocompatible and degradable, but their mechanical strength and thermal stability are poor. Synthetic polymers have ideal mechanical properties but poor osseointegration.	[86,87,88,89,90,91,92]

## 4. Preparation of Bioink for 3D Printed Scaffolds

Nanomaterials are incorporated into 3D printed scaffolds in two different steps: the bioink is prepared by mixing substances during the manufacturing process (pre-filling, PF), and then the 3D printing is continued [93,94]. Alternatively, in the final step of printing, molecules are loaded by immersing the 3D printed scaffolds in solution (direct loading, DL) [95]. PF method is often suitable for 3D printed scaffolds that can be delivered and applied in real time; however, antibiotics such as the cephalosporin family are significantly less efficient when heated. Therefore, when the 3D printing process is carried out at high temperature and pressure, the DL method should be used to apply to sensitive molecules. This section focuses on bioinks for application to PF [96].

### 4.1. Evaluation Parameters of Bioink

The key to pre-loading bioink for nanomaterials is to find a balance among printability, biocompatibility and mechanical properties [97]. Printability is the conversion of the formability of bioink, including adjustable biomaterial viscosity, rapid changeover properties from sol to gel, and printable process parameters. Biocompatibility requires that bioinks resemble the cellular microenvironment in the human body as closely as possible, allowing cells to proliferate, expand, differentiate and interact with each other. Mechanical properties require gelatinous bioinks to be mechanically strong to support subsequent culture and implantation processes. Bioprint structures often need to be cultured in vitro, often accompanied by perfusion and degradation of nutrients, requiring considerable strength support. Insufficient mechanical properties can also lead to transplant failure.

### 4.2. Classification of Bioinks

Based on curing methods, bioinks can be classified into four main types, namely ion-crosslinked, temperature-sensitive, photosensitive, and shear-thinning.

The ion crosslinking type is mainly to achieve the curing of the hydrogels through the ion crosslinking reactions, such as alginate series inks. The sodium ions in sodium alginate are replaced with calcium ions to obtain calcium alginate hydrogels [98,99]. Temperature-sensitive inks are mainly heated or cooled to realize the transformation of the ink from sol state to gel state, such as gelatin inks, printing needs to heat the nozzle to melt gelatin and cool the bottom of the printing platform to achieve gelatin shaping [100]. Photosensitive inks are mainly used to activate the photoinitiator in the ink by light to realize the transformation of the ink from a sol to a gel, such as methacrylic anhydride gelatin (GelMA, EFL-GM series) materials [101]. Shear-thinning inks mainly use the phenomenon that the apparent viscosity of some materials decreases with the increase in shear stress, which appears as a gel state when not subjected to shear force, and becomes a sol when subjected to shear force, such as the formation of bioinks with carbomer glue + GelMA or collagen [102].

### 4.3. Components of Bioinks

The extracellular matrix secreted by cells is mainly composed of three major components: structural proteins (collagen, elastin, etc.), specific proteins (fibrin, etc.) and proteoglycans. The ideal bioink is undoubtedly close to the natural extracellular matrix, so the bioinks required by different cells also needs to be adjusted, in principle, to add as much as possible the substances required for cell growth in vivo. At present, the most used bioinks are alginate-based bioinks, collagen bioinks and GelMA materials.

Alginate-based bioinks have good forming performance and mechanical properties, but have the disadvantage of weak biocompatibility, which directly affects the transformation of cells into tissues after printing [103]. Collagen bioinks have good biocompatibility due to their animal origin, but have the disadvantages of slow forming speed and poor mechanical properties, requiring subsequently modification or mixing into other materials [104]. GelMA materials have the advantages of both printability and formability, expected to be widely used in bio-3D printing [105].

By increasing the concentration of hydrogel and the density of crosslinker, the curing time can be accelerated, the strength of the hydrogel can be improved, and it is conducive to better printing and forming. At the same time, it will also reduce the water content of the gel and the micropore size inside the gel, which is not conducive to cell survival and the deposition of extracellular matrix. There is therefore a suitable range of forming processes for bioinks, which is known as the Biofabrication window [105,106]. This window mainly refers to a successful printing interval that considers the printing requirements, cell viability and growth requirements, and combines the printing process parameters (such as printing speed, extrusion pressure, printing temperature, ink concentration, crosslinker concentration, light curing time, etc.). During the specific printing process, the gel properties can be adjusted by moving the window in different directions, according to the actual cell type and printing accuracy.

## 5. 3D Printed Scaffolds Loaded with Nanomaterials

In order to achieve the desired effect, post-printing modifications are often used to improve scaffolding performance. These post-processing steps can encompass various techniques, including surface modifications (e.g., depositing hydroxyapatite onto 3D-printed gels to enhance osteoinductivity) [107] and thermal treatments like high-temperature degreasing and sintering for ceramic parts. Such modifications not only improve the differentiation and growth of stem cells in vitro, but also improves histocompatibility after transplantation [108]. Nanomaterials are materials whose one-dimensional structures are at least nanoscale or consist of nano-structural units with special properties. It has been extensively studied in the field of tissue engineering, used as a scaffold for cell growth to promote bone tissue regeneration and nerve repair [42]. Nanomaterials can be used for diagnosing diseases through bioimaging by spectroscopic and fluorescent signals [109]. At the same time, nanomaterials can also be used as biosensors to effectively monitor disease progression. Nanomaterials can offer many unique potential functions for the targeted therapy of diseases [110]. This section focuses on the targeted transport capabilities of 3D-printed scaffold loaded nanoparticles in the field of bone defects.

### 5.1. Exosome

Exosomes are small membrane vesicles (30–150 nm) that contain complex RNAs and proteins, and today, disk-shaped vesicles with a diameter of 40–100 nm. Exosomes were first discovered in sheep reticulocytes in 1983 and were named by Johnston in 1987. A variety of cells secrete exosomes in both normal and pathological conditions. It is mainly derived from the polyvesicular body formed by the invagination of intracellular lysosomal granules, which is released into the extracellular matrix after fusion with the cell membrane by the polyvesicular extracorporeal membrane [111]. All cell types can secrete exosomes, and it is also present in body fluids, including blood, saliva, urine, cerebrospinal fluid, and breast milk. Precise molecular mechanisms regarding their secretion, uptake, composition, “carriers” and corresponding functions are just beginning to be studied [112]. Exosomes are regarded as membrane vesicles specifically secreted, involved in intercellular communication, and there is growing interest in the study of exosomes, whether to study their function or to understand how to use them in the development of minimally invasive diagnosis [113].

Due to the lack of vascularization of newly formed bone, large segment bone regeneration remains a significant challenge. Conventional strategies primarily combine bone scaffolds with seed cells and growth factors to regulate osteogenesis and angiogenesis. Studies have used a cell-free tissue engineering system to replace seed cells with functional exosomes. ATDC5-derived exosomes (the size and size distribution was 114.2 ± 1.8 nm) encapsulate the VEGF gene to construct engineered exosomes. Specific exosome-anchored peptide CP05 acts as a hymen, effectively binding exosome nanoparticles to 3D-printed porous bone scaffolds. The results show that engineered exosomes have a dual role in inducing osteogenic differentiation of mesenchymal stem cells while also releasing VEGF genes to reshape the vascular system [114].

### 5.2. Liposome and Micelle

Liposome is an artificial membrane. The phospholipid molecule hydrophilic head is inserted into the water, the liposome hydrophobic tail extends to the air, and after agitation forms a spherical liposome of the bilayer lipid molecule, with a diameter ranging from 25 to 1000 nm [115]. Liposomes can be used for genetic modification, or for the preparation of drugs, taking advantage of the fact that liposomes can fuse with cell membranes to deliver drugs into cells. A micelle is a molecule whose lipophilic tail ends are clustered within the micelle to avoid contact with polar water molecules; The polar hydrophilic tip of the molecule is exposed to the outside, interacts with the polar water molecules, and has a protective effect on the interior of the micelle. The compound forming the micelle is generally a bipolar molecule, so micelles can be soluble in polar solvents such as water, and can also be dissolved in non-polar solvents in the form of anti-micelles [116].

Aspirin has been shown to have a protective effect on bones. However, drug resistance and its cytotoxicity significantly limit its clinical application. Sustained-release systems have been shown to delay the release, absorption, metabolism, and excretion of drugs in the body, prolonging the drug’s duration of action and mitigating side effects. The study found that the aspirin-rich liposome delivery system (Asp@Lipo) significantly promoted osteogenesis and immunomodulation of human mesenchymal stem cells (hMSCs). Bioactive composite (PCL-Asp@Lipo) scaffolds were then prepared based on polycaprolactone (PCL), confirmed by in vivo and in vitro experiments to enhance osteogenic differentiation and exhibit more osteogenic activity [117]. Simvastatin (SMV) is a hydrophobic drug that has shown potential for osteogenesis stimulation. Hydrophobic drugs are difficult to establish in a hydrophilic polymer matrix through stable loads of physical interactions. Researchers loaded the SMV into a polymer micelle of PLA-PEG-PLA and mixed it with N-methacryloyl chitosan and PEG dimethacrylate to prepare a hydrogel by 3D printing. This method improved the mechanical properties of the hydrogel, providing sufficient mechanical support while maintaining the biodegradability and biocompatibility of the material [118].

### 5.3. Inorganic Nanoparticles

Inspired by the natural composition of bone tissue, composites made from inorganic phases such as nano-silicate particles, calcium phosphate and bioactive glass, combined with biopolymer matrices, have been studied as building blocks for bio-fabrication of bone structures. In addition to elements that mimic the physiological structure of bone, these inorganic/organic composites can also be designed with specific cohesive, rheological and mechanical properties, with both inorganic and organic components contributing to the bioactivity of the composites [3].

The researchers developed a novel hierarchical bio-functionalized 3D printed porous Ti6Al4V scaffold with enhanced osteointegration. This study built a biomimetic extracellular matrix (ECM) at the micron scale within the interconnect pores of the scaffold. At the nanoscale, the drug icariin@Mg-MOF-74 was encased in an ECM-like structure to further control the release of icariin and Mg^2+^. In vivo experiments have demonstrated that implantation of biofunctionalized scaffolds into the distal femur of osteoporosis rats within the bone marrow can form abundant new bone and improve osteointegration [119].

### 5.4. Dendrimers

Dendrimers are macromolecules formed by repeated and linear connections of oligomers through dendritic units, with dendritic structure. They typically consist of a kernel, polymer backbone, and side chains of the branch units. Dendrimers contain a cavity structure, which surface is enriched with active functional groups, with controllable physical and chemical properties. The most common materials are polyamidoamine (PAMAM), polylysine (poly(L-lysine), PLL), polyethylenimine (PEI), polyacrylamine (propyleneimine, PPI) [120]. However, most dendrimers are non-degradable and carry a risk of accumulation in the body as drug delivery systems. Some dendrimers have many terminal amino groups on the surface of their three-dimensional spheres. Too many terminal amino groups can lead to stronger cationicity. Too strong cationic is easy to cause cell membrane rupture or even apoptosis, which is the cytotoxicity of dendrimers. Unlike liposomes, nanoparticles, etc., that control particle size through physical means, dendrimers control particle size and morphology through chemical synthesis.

There have been studies to prepare dendrimer particles (DPs) using BMP-2 plasmids encapsulated by polyamidine (G4-PAMAM). In order to create a suitable environment for the growth and differentiation of stem cells, polyol-lactic acid (PLLA) and polyol-lactic acid/polyethylene oxide (PLLA/PEO) scaffolds containing nHA and DPs were prepared. The results showed that the presence of HA and DP in PLLA/PEO stents can promote osteogenic differentiation of hASCs [121]. In general, dendrimers have the potential to become the mainstream drug delivery technology. Its superiority will attract more scientists to study the delivery system related materials, preparation technology, delivery methods, delivery routes and so on.

A diverse array of materials loaded onto 3D-printed scaffolds continue to emerge. Table 2 summarizes the nanomaterials loaded onto 3D-printed scaffolds that have been applied for bone defect repair over the past five years.

## 6. Challenges and Prospects

Since the development of 3D printing, many technical breakthroughs and progress have been made. Especially in recent years, 3D printing has been widely promoted and popularized by many factors. LAB and SLA represent the future development trend of 3D micro-nanometer printing, which can provide new methods for the structural manufacturing of some new materials and expand the application and development of functional materials. Other technologies will also complement the original foundation with functions to obtain higher molding accuracy. However, there are still some problems in 3D nano-printing that need further exploration and research.

First, the high cost of equipment and materials, combined with scalability limitations, hinders the widespread adoption of this technology in industrial and academic settings. Current bioprinters, especially those capable of high precision, are often very expensive (over 100,000 per unit), with industrial-grade laser sintering and FDM models also costing hundreds of thousands. While accuracy and molding quality are high, the production time for a single piece remains long, hindering large-scale manufacturing and clinical translation. Second, the limited availability and slow development of suitable materials restrict the range of applications. Although a wide range of basic materials are available, there is a significant lack of bioinks and printable materials specifically optimized for complex biological functions, tissue-specific properties, and in vivo applications. Developing functional materials that meet stringent biocompatibility, biodegradability, and mechanical property requirements for bone tissue engineering remains a critical bottleneck. Finally, significant challenges remain in achieving precise control over multi-scale and multi-material printing, which is essential for creating complex functional devices with heterogeneous properties. Achieving both nanoscale precision and macroscopic composite structures simultaneously with current printing devices is technically challenging. The development of multi-material printing devices capable of integrating diverse materials with varied properties at different scales remains an area requiring substantial innovation.

The future of nano-based 3D printing in bone tissue engineering is promising, with several key directions for advancement. Addressing the current challenges will involve several prioritizing critical areas. First, develop novel, cost-effective printing platforms that can achieve high throughput and scalability without compromising precision. This includes exploring hybrid printing technologies and automation to streamline the fabrication process. Second, drive innovation in advanced bioinks and functional materials. Future research will focus on engineering smart bioinks with active biological components (e.g., growth factors, cells, nano-drugs) that can precisely mimic the native extracellular matrix and actively promote osteogenesis and angiogenesis. This also involves designing materials with tunable mechanical properties and controlled degradation rates. Third, advance multi-scale and multi-material printing capabilities. The ability to simultaneously incorporate micro- and nano-features from diverse materials within a single scaffold will be crucial for creating biomimetic constructs that closely replicate bone’s complex hierarchical structure. This includes integrating vascularization strategies and guided cell migration cues directly into the scaffold design. Finally, enhance computational design and AI integration. Leveraging artificial intelligence and machine learning for optimized scaffold design, material selection, and process parameter prediction will significantly accelerate development and improve outcomes. By overcoming these hurdles, nano-based 3D printing is poised to revolutionize personalized bone regeneration, leading to the creation of truly biomimetic and functional bone implants.

## Figures and Tables

**Table 2:** Summary of 3D printed scaffold loaded nanomaterials for bone defects over the last five years.

Printing Technology	Nanomaterial	Scaffold Composition	Application In Vitro/In Vivo	Characteristic	Reference
BJ	nano-sized micelle	ß-TCP	hFOBs, MG-63, HUVECs	sequential drug release, improvement in osteoblast proliferation and endothelial formation, bacterial inhibition, guiding better bone regeneration for post-traumatic defect repair	[122]
DIW	CNC, nHA	gelatin	MG-63	enhancing compressive modulus in both dry and wet states, suitability for non-load-bearing or low-load-bearing bone tissue applications, improving the viability and adhesion of osteoblast-like cells	[123]
	calcium titanate nanoparticles	alginate	rats	osteogenic, angiogenic, synergistic effect of the scaffold’s osteoconductive properties and MSCs’ regenerative potential, bone regeneration	[124]
	Ox-gCN	alginate/gelatin	hBMSCs, RAW 264.7, rats	facilitating M2 macrophages polarization, inducing cells differentiation, biofilm reduction efficacy against Gram-negative and positive bacteria, osteogenesis, hemostatic ability, anti-inflammatory, osteo-immunomodulatory, for traumatic bone injury repair	[125]
	amorphous magnesium phosphate-graphene oxide nano particles	chitosan/glycerol phosphate	MG-63, MSCs, rats	improving compressive strengths, enhancing antibacterial activity, increasing MSCs activity (adhesion, viability, proliferation and osteogenic differentiation), improving osteogenic ability in vivo	[126]
	NP@Eth nanoparticles	SA/GelMA	BMSCs, rats	blocking sympathetic nervous system activation, promoting the osteogenic differentiation and migration of BMSCs, inhibiting osteoclastogenesis, improving bone regeneration	[127]
	Calcium silicate nanowires	alginate	rabbit BMSCs	leading cells to ordered alignment and improving differentiation	[128]
	polydopamine nanoparticles	alginate, tempo-oxidized cellulose nanofibrils	MC3T3-E1	inducing osteogenesis	[129]
	nano attapulgite	SA/gelatin	mouse BMSCs, rabbits	mineralization, biocompatibility, promoting osteogenesis	[130]
DIW, FDM	bMSN, MgO nanoparticles	GelMA/GGMA/PCL	rat BMSCs, HUVECs, rats	cytocompatibility, stimulating angiogenic behavior and osteogenic differentiation, enhancing vascularization, promoting bone regeneration	[131]
DLP	Mn single-atom nanozyme	BG + BCP	hBMSCs, rabbits	synergetic strategy of chemodynamic therapy (CDT)/SDT enhancing antibacterial activity and bone regeneration	[132]
EBM	UiO-66 nanocrystals	Ti6Al4V	BMSCs, HUVECs, rabbits	osteogenic and angiogenic induction, promoting intercellular crosstalk by enhancing paracrine effects	[133]
FDM	collagen type I-nHA matrix	PCE20kC	MC3T3-E1, endothelial cells	interconnected multi-scale pores, a compressive modulus comparable to cancellous bone, promoting osteoblast proliferation, differentiation and mineralization, vascularization	[134]
	LPA nanoparticles	PLGA/PCL	MC3T3-E1, mice, rats	enhancing cells activity (attachment, proliferation, osteogenic differentiation and mineralization), cytocompatibility, cell recruitment ability, promoting bone regeneration	[135]
	nHA, Sr-nHA	PLLA/PCL/PHBV	MC3T3-E1	biocompatible, high proliferation capacity, osteoinductive	[136]
	black phosphorus nanosheets with DNA	PCL	HUVECs, MSCs, rats	promoting the growth of mature blood vessels, inducing osteogenesis, promoting new bone formation	[137]
	nano tantalum	PLA/β-TCP	MC3T3-E1	promoting cell proliferation and migration, inducing osteogenesis	[31]
	BreSr	PLA/PCL	human osteoblasts, rats	supporting viability and proliferation, regenerating bone tissue	[89]
	nHA-EGCG nanoparticles	PCLA	MC3T3-E1, HepG2, A549	stimulating osteogenic differentiation, in situ antibacterial ability, promoting cell adhesion	[138]
	Chit@IOC	PCL	rat MSCs, rats	ultrahigh inorganic content, high resilience, multiple-therapeutics delivery, cellular activation	[139]
LPBF	Mg-Al LDH nanosheets	Mg	hBMSCs, HUVECs, rabbits	augmenting bioactivity and osteointegration	[140]
sacrificial templating	nano-silica	gelatin/PLGA	HUVECs, hMSCs	surface micropatterning, improving cell growth (orientation adhesion and growth with a certain direction)	[141]
SLM	ICA@MOF	Ti6Al4V	Raw264.7, BMMs, rat BMSCs	facilitating cell adhesion, enhancing biocompatibility, promoting new bone formation, improving the polarization of M0 macrophages to M2-type, inducing the secretion of anti-inflammatory cytokines	[119]

Abbreviations: A549 = lung adenocarcinoma cells; BCP = biphasic calcium phosphate; BG = bioglass; BJ = binder jetting; BMMs = mouse bone marrow macrophages; BMSCs = bone marrow mesenchymal stem cells; bMSN = biodegradable mesoporous silica nanoparticles; BreSr = strontium (Sr) doped bredigite (Bre) nanoparticles; Chit@IOC = nanohybrids composed of bioactive inorganic nanoparticle core (hydroxyapatite, bioactive glass, or mesoporous silica) and chitosan shell; CNC = cellulose nanocrystals; DIW = direct ink writing; DLP = digital light processing; EBM = electron beam melting; EGCG = epigallocatechin-3-gallate; FDM = fused deposition modeling; GelMA = methacrylated gelatin; GGMA = methacrylated gellan gum; HepG2 = hepatocellular carcinoma cells; hFOBs = human fetal osteoblasts; HUVECs = human umbilical vein endothelial cells; ICA@MOF = an icariin-containing MOF solid; LDH = layered double hydroxides; LPA = lysophosphatidic acid; LPBF = laser powder bed fusion; MC3T3-E1 = mouse embryonic osteoblast precursor cells; MG-63 = osteosarcoma cells; MgO = magnesium oxide; MOF = metal-organic framework; nHA = nano-hydroxyapatite; NP@Eth =nifedipine-loaded ethosome; Ox-gCN = a highly oxidized two-dimensional graphitic carbon nitride as a nano-photocatalyst; PCE20kC = poly(ε-caprolactone)-polyethylene glycol 20k-poly(ε-caprolactone); PCL = polycaprolactone; PCLA = polymerization of caprolactone and lactide; PHBV = poly(3-hydroxybutyrate-co-3-hydroxyvalerate); PLA = polylactic acid; PLGA = poly lactic-co-glycolic acid; PLLA = polymeric blends of poly-L-lactic acid; RAW 264.7 = mouse macrophage cells; SA = sodium alginate; SLM = selective laser melting; Sr-nHA = strontium-substituted nano-HA; ß-TCP = beta-tricalcium phosphate; UiO-66 = a MOF, synthesized using a zirconium oxide complex bridged by 1,4-benzene dicarboxylic acid ligands.

## Data Availability

No new data were created.

## References

[1] Gil L.F., Nayak V.V., Benalcázar Jalkh E.B., Tovar N., Chiu K.J., Salas J.C., Marin C., Bowers M., Freitas G., Mbe Fokam D.C. (2022). Laddec^®^ versus Bio-Oss^®^: The effect on the healing of critical-sized defect—Calvaria rabbit model. J. Biomed. Mater. Res. B Appl. Biomater..

[2] Li M., Sun D., Zhang J., Wang Y., Wei Q., Wang Y. (2022). Application and development of 3D bioprinting in cartilage tissue engineering. Biomater. Sci..

[3] van der Heide D., Cidonio G., Stoddart M.J., D’Este M. (2022). 3D printing of inorganic-biopolymer composites for bone regeneration. Biofabrication.

[4] Feng B., Zhang M., Qin C., Zhai D., Wang Y., Zhou Y., Chang J., Zhu Y., Wu C. (2023). 3D printing of conch-like scaffolds for guiding cell migration and directional bone growth. Bioact. Mater..

[5] Wang B., Feng C., Liu Y., Mi F., Dong J. (2022). Recent advances in biofunctional guided bone regeneration materials for repairing defective alveolar and maxillofacial bone: A review. Jpn. Dent. Sci. Rev..

[6] Xue N., Ding X., Huang R., Jiang R., Huang H., Pan X., Min W., Chen J., Duan J.A., Liu P. (2022). Bone Tissue Engineering in the Treatment of Bone Defects. Pharmaceuticals.

[7] Sun K., Li R., Jiang W., Sun Y., Li H. (2016). Comparison of three-dimensional printing and vacuum freeze-dried techniques for fabricating composite scaffolds. Biochem. Biophys. Res. Commun..

[8] Hettiarachchi K., Lee A.P. (2010). Polymer-lipid microbubbles for biosensing and the formation of porous structures. J. Colloid Interface Sci..

[9] Samadi A., Salati M.A., Safari A., Jouyandeh M., Barani M., Singh Chauhan N.P., Golab E.G., Zarrintaj P., Kar S., Seidi F. (2022). Comparative review of piezoelectric biomaterials approach for bone tissue engineering. J. Biomater. Sci. Polym. Ed..

[10] Li Y., Zhang X., Lin J., Li R., Yue T. (2019). Extracting lipid vesicles from plasma membranes via self-assembly of clathrin-inspired scaffolding nanoparticles. Colloids Surf. B Biointerfaces.

[11] Kodama H. (1981). Automatic method for fabricating a three-dimensional plastic model with photo-hardening polymer. Rev. Sci. Instrum..

[12] Herbert A.J. (1982). Solid Object Generation. J. Appl. Photogr. Eng..

[13] Kumar H., Kim K. (2020). Stereolithography 3D Bioprinting. Methods Mol. Biol..

[14] Yu C., Schimelman J., Wang P., Miller K.L., Ma X., You S., Guan J., Sun B., Zhu W., Chen S. (2020). Photopolymerizable Biomaterials and Light-Based 3D Printing Strategies for Biomedical Applications. Chem. Rev..

[15] Kotz F., Risch P., Helmer D., Rapp B.E. (2019). High-Performance Materials for 3D Printing in Chemical Synthesis Applications. Adv. Mater..

[16] Melchels F.P., Feijen J., Grijpma D.W. (2010). A review on stereolithography and its applications in biomedical engineering. Biomaterials.

[17] Kafle A., Luis E., Silwal R., Pan H.M., Shrestha P.L., Bastola A.K. (2021). 3D/4D Printing of Polymers: Fused Deposition Modelling (FDM), Selective Laser Sintering (SLS), and Stereolithography (SLA). Polymers.

[18] Della Bona A., Cantelli V., Britto V.T., Collares K.F., Stansbury J.W. (2021). 3D printing restorative materials using a stereolithographic technique: A systematic review. Dent. Mater..

[19] Tian Y., Chen C., Xu X., Wang J., Hou X., Li K., Lu X., Shi H., Lee E.S., Jiang H.B. (2021). A Review of 3D Printing in Dentistry: Technologies, Affecting Factors, and Applications. Scanning.

[20] Li X., Liu B., Pei B., Chen J., Zhou D., Peng J., Zhang X., Jia W., Xu T. (2020). Inkjet Bioprinting of Biomaterials. Chem. Rev..

[21] Xu Y., Zhang F., Zhai W., Cheng S., Li J., Wang Y. (2022). Unraveling of Advances in 3D-Printed Polymer-Based Bone Scaffolds. Polymers.

[22] Zhou X., Esworthy T., Lee S.J., Miao S., Cui H., Plesiniak M., Fenniri H., Webster T., Rao R.D., Zhang L.G. (2019). 3D Printed scaffolds with hierarchical biomimetic structure for osteochondral regeneration. Nanomedicine.

[23] Malinauskas M., Zukauskas A., Hasegawa S., Hayasaki Y., Mizeikis V., Buividas R., Juodkazis S. (2016). Ultrafast laser processing of materials: From science to industry. Light-Sci. Appl..

[24] Tappa K., Jammalamadaka U. (2018). Novel Biomaterials Used in Medical 3D Printing Techniques. J. Funct. Biomater..

[25] Parulski C., Jennotte O., Lechanteur A., Evrard B. (2021). Challenges of fused deposition modeling 3D printing in pharmaceutical applications: Where are we now?. Adv. Drug Deliv. Rev..

[26] Wasti S., Adhikari S. (2020). Use of Biomaterials for 3D Printing by Fused Deposition Modeling Technique: A Review. Front. Chem..

[27] Bandari S., Nyavanandi D., Dumpa N., Repka M.A. (2021). Coupling hot melt extrusion and fused deposition modeling: Critical properties for successful performance. Adv. Drug Deliv. Rev..

[28] Cailleaux S., Sanchez-Ballester N.M., Gueche Y.A., Bataille B., Soulairol I. (2021). Fused Deposition Modeling (FDM), the new asset for the production of tailored medicines. J. Control. Release.

[29] Melocchi A., Uboldi M., Maroni A., Foppoli A., Palugan L., Zema L., Gazzaniga A. (2020). 3D printing by fused deposition modeling of single- and multi-compartment hollow systems for oral delivery—A review. Int. J. Pharm..

[30] Karimipour-Fard P., Jeffrey M.P., JonesTaggart H., Pop-Iliev R., Rizvi G. (2021). Development, processing and characterization of Polycaprolactone/Nano-Hydroxyapatite/Chitin-Nano-Whisker nanocomposite filaments for additive manufacturing of bone tissue scaffolds. J. Mech. Behav. Biomed. Mater..

[31] Liu T., Li B., Chen G., Ye X., Zhang Y. (2022). Nano tantalum-coated 3D printed porous polylactic acid/beta-tricalcium phosphate scaffolds with enhanced biological properties for guided bone regeneration. Int. J. Biol. Macromol..

[32] Tharakan S., Khondkar S., Ilyas A. (2021). Bioprinting of Stem Cells in Multimaterial Scaffolds and Their Applications in Bone Tissue Engineering. Sensors.

[33] Irvine S.A., Venkatraman S.S. (2016). Bioprinting and Differentiation of Stem Cells. Molecules.

[34] Derakhshanfar S., Mbeleck R., Xu K., Zhang X., Zhong W., Xing M. (2018). 3D bioprinting for biomedical devices and tissue engineering: A review of recent trends and advances. Bioact. Mater..

[35] Hölzl K., Lin S., Tytgat L., Van Vlierberghe S., Gu L., Ovsianikov A. (2016). Bioink properties before, during and after 3D bioprinting. Biofabrication.

[36] Filardo G., Petretta M., Cavallo C., Roseti L., Durante S., Albisinni U., Grigolo B. (2019). Patient-specific meniscus prototype based on 3D bioprinting of human cell-laden scaffold. Bone Jt. Res..

[37] Nganga S., Moritz N., Kolakovic R., Jakobsson K., Nyman J.O., Borgogna M., Travan A., Crosera M., Donati I., Vallittu P.K. (2014). Inkjet printing of Chitlac-nanosilver—A method to create functional coatings for non-metallic bone implants. Biofabrication.

[38] Hakobyan D., Kerouredan O., Remy M., Dusserre N., Medina C., Devillard R., Fricain J.C., Oliveira H. (2020). Laser-Assisted Bioprinting for Bone Repair. Methods Mol. Biol..

[39] Ong C.S., Yesantharao P., Huang C.Y., Mattson G., Boktor J., Fukunishi T., Zhang H., Hibino N. (2018). 3D bioprinting using stem cells. Pediatr. Res..

[40] Koch L., Gruene M., Unger C., Chichkov B. (2013). Laser assisted cell printing. Curr. Pharm. Biotechnol..

[41] Kérourédan O., Hakobyan D., Rémy M., Ziane S., Dusserre N., Fricain J.C., Delmond S., Thébaud N.B., Devillard R. (2019). In situ prevascularization designed by laser-assisted bioprinting: Effect on bone regeneration. Biofabrication.

[42] Li J., Chen M., Fan X., Zhou H. (2016). Recent advances in bioprinting techniques: Approaches, applications and future prospects. J. Transl. Med..

[43] Kačarević Ž.P., Rider P.M., Alkildani S., Retnasingh S., Smeets R., Jung O., Ivanišević Z., Barbeck M. (2018). An Introduction to 3D Bioprinting: Possibilities, Challenges and Future Aspects. Materials.

[44] Ashammakhi N., Hasan A., Kaarela O., Byambaa B., Sheikhi A., Gaharwar A.K., Khademhosseini A. (2019). Advancing Frontiers in Bone Bioprinting. Adv. Healthc. Mater..

[45] Su X., Wang T., Guo S. (2021). Applications of 3D printed bone tissue engineering scaffolds in the stem cell field. Regen. Ther..

[46] Touya N., Devun M., Handschin C., Casenave S., Ahmed Omar N., Gaubert A., Dusserre N., De Oliveira H., Kérourédan O., Devillard R. (2022). In vitroandin vivocharacterization of a novel tricalcium silicate-based ink for bone regeneration using laser-assisted bioprinting. Biofabrication.

[47] da Costa Valente M.L., de Oliveira T.T., Kreve S., Batalha R.L., de Oliveira D.P., Pauly S., Bolfarini C., Bachmann L., Dos Reis A.C. (2021). Analysis of the mechanical and physicochemical properties of Ti-6Al-4 V discs obtained by selective laser melting and subtractive manufacturing method. J. Biomed. Mater. Res. B Appl. Biomater..

[48] Gokuldoss P.K., Kolla S., Eckert J. (2017). Additive Manufacturing Processes: Selective Laser Melting, Electron Beam Melting and Binder Jetting-Selection Guidelines. Materials.

[49] Xu J.Y., Chen X.S., Zhang C.Y., Liu Y., Wang J., Deng F.L. (2016). Improved bioactivity of selective laser melting titanium: Surface modification with micro-/nano-textured hierarchical topography and bone regeneration performance evaluation. Mater. Sci. Eng. C Mater. Biol. Appl..

[50] Cui J., Yi Y., Zhang J., Chai L., Jin H. (2022). Preparation and mechanical properties analysis of porous structure for bone tissue engineering. Biomed. Mater. Eng..

[51] Wang W., Wang Z., Fu Y., Dunne N., Liang C., Luo X., Liu K., Li X., Pang X., Lu K. (2020). Improved osteogenic differentiation of human amniotic mesenchymal stem cells on gradient nanostructured Ti surface. J. Biomed. Mater. Res. A.

[52] Maher S., Wijenayaka A.R., Lima-Marques L., Yang D., Atkins G.J., Losic D. (2021). Advancing of Additive-Manufactured Titanium Implants with Bioinspired Micro- to Nanotopographies. ACS Biomater. Sci. Eng..

[53] Cao N.J., Zhu Y.H., Gao F., Liang C., Wang Z.B., Zhang Y., Hao C.P., Wang W. (2021). Gradient nanostructured titanium stimulates cell responses in vitro and enhances osseointegration in vivo. Ann. Transl. Med..

[54] Bloise N., Waldorff E.I., Montagna G., Bruni G., Fassina L., Fang S., Zhang N., Jiang J., Ryaby J.T., Visai L. (2022). Early Osteogenic Marker Expression in hMSCs Cultured onto Acid Etching-Derived Micro- and Nanotopography 3D-Printed Titanium Surfaces. Int. J. Mol. Sci..

[55] Zhang Y., Sun N., Zhu M., Qiu Q., Zhao P., Zheng C., Bai Q., Zeng Q., Lu T. (2022). The contribution of pore size and porosity of 3D printed porous titanium scaffolds to osteogenesis. Biomater. Adv..

[56] Ren B., Wan Y., Liu C., Wang H., Yu M., Zhang X., Huang Y. (2021). Improved osseointegration of 3D printed Ti-6Al-4V implant with a hierarchical micro/nano surface topography: An in vitro and in vivo study. Mater. Sci. Eng. C Mater. Biol. Appl..

[57] Xiang Gu G., Su I., Sharma S., Voros J.L., Qin Z., Buehler M.J. (2016). Three-Dimensional-Printing of Bio-Inspired Composites. J. Biomech. Eng..

[58] Willson K., Atala A., Yoo J.J. (2021). Bioprinting Au Natural: The Biologics of Bioinks. Biomolecules.

[59] Mahendiran B., Muthusamy S., Sampath S., Jaisankar S.N., Popat K.C., Selvakumar R., Krishnakumar G.S. (2021). Recent trends in natural polysaccharide based bioinks for multiscale 3D printing in tissue regeneration: A review. Int. J. Biol. Macromol..

[60] Boga J.C., Miguel S.P., de Melo-Diogo D., Mendonça A.G., Louro R.O., Correia I.J. (2018). In vitro characterization of 3D printed scaffolds aimed at bone tissue regeneration. Colloids Surf. B Biointerfaces.

[61] Wang X., Cao Y., Jing L., Chen S., Leng B., Yang X., Wu Z., Bian J., Banjerdpongchai R., Poofery J. (2021). Three-Dimensional RAW264.7 Cell Model on Electrohydrodynamic Printed Poly(ε-Caprolactone) Scaffolds for In Vitro Study of Anti-Inflammatory Compounds. ACS Appl. Bio Mater..

[62] Diogo G.S., Marques C.F., Freitas-Ribeiro S., Sotelo C.G., Pérez-Martin R.I., Pirraco R.P., Reis R.L., Silva T.H. (2022). Mineralized collagen as a bioactive ink to support encapsulation of human adipose stem cells: A step towards the future of bone regeneration. Biomater. Adv..

[63] Mondal D., Haghpanah Z., Huxman C.J., Tanter S., Sun D., Gorbet M., Willett T.L. (2021). mSLA-based 3D printing of acrylated epoxidized soybean oil-nano-hydroxyapatite composites for bone repair. Mater. Sci. Eng. C Mater. Biol. Appl..

[64] Benavides-Guerrero J.A., Gerlein L.F., Trudeau C., Banerjee D., Guo X., Cloutier S.G. (2022). Synthesis of vacancy-rich titania particles suitable for the additive manufacturing of ceramics. Sci. Rep..

[65] Tselikos G., Rasul S., Groen P., Li C., Khaliq J. (2021). In Situ Printing and Functionalization of Hybrid Polymer-Ceramic Composites Using a Commercial 3D Printer and Dielectrophoresis-A Novel Conceptual Design. Polymers.

[66] Martinez J.S., Peterson S., Hoel C.A., Erno D.J., Murray T., Boyd L., Her J.-H., McLean N., Davis R., Ginty F. (2022). High resolution DLP stereolithography to fabricate biocompatible hydroxyapatite structures that support osteogenesis. PLoS ONE.

[67] Zhang F., Spies B.C., Willems E., Inokoshi M., Wesemann C., Cokic S.M., Hache B., Kohal R.J., Altmann B., Vleugels J. (2022). 3D printed zirconia dental implants with integrated directional surface pores combine mechanical strength with favorable osteoblast response. Acta Biomater..

[68] Zhu Y., Liu K., Deng J., Ye J., Ai F., Ouyang H., Wu T., Jia J., Cheng X., Wang X. (2019). 3D printed zirconia ceramic hip joint with precise structure and broad-spectrum antibacterial properties. Int. J. Nanomed..

[69] Quodbach J., Bogdahn M., Breitkreutz J., Chamberlain R., Eggenreich K., Elia A.G., Gottschalk N., Gunkel-Grabole G., Hoffmann L., Kapote D. (2022). Quality of FDM 3D Printed Medicines for Pediatrics: Considerations for Formulation Development, Filament Extrusion, Printing Process and Printer Design. Ther. Innov. Regul. Sci..

[70] Piedra-Cascón W., Krishnamurthy V.R., Att W., Revilla-León M. (2021). 3D printing parameters, supporting structures, slicing, and post-processing procedures of vat-polymerization additive manufacturing technologies: A narrative review. J. Dent..

[71] Govender R., Kissi E.O., Larsson A., Tho I. (2021). Polymers in pharmaceutical additive manufacturing: A balancing act between printability and product performance. Adv. Drug Deliv. Rev..

[72] Zhu Y., Cao N., Zhang Y., Cao G., Hao C., Liu K., Li X., Wang W. (2022). The Ability and Mechanism of nHAC/CGF in Promoting Osteogenesis and Repairing Mandibular Defects. Nanomaterials.

[73] Petretta M., Gambardella A., Boi M., Berni M., Cavallo C., Marchiori G., Maltarello M.C., Bellucci D., Fini M., Baldini N. (2021). Composite Scaffolds for Bone Tissue Regeneration Based on PCL and Mg-Containing Bioactive Glasses. Biology.

[74] Marycz K., Smieszek A., Targonska S., Walsh S.A., Szustakiewicz K., Wiglusz R.J. (2020). Three dimensional (3D) printed polylactic acid with nano-hydroxyapatite doped with europium(III) ions (nHAp/PLLA@Eu(3+)) composite for osteochondral defect regeneration and theranostics. Mater. Sci. Eng. C Mater. Biol. Appl..

[75] Velásquez-García L.F., Kornbluth Y. (2021). Biomedical Applications of Metal 3D Printing. Annu. Rev. Biomed. Eng..

[76] Putra N.E., Mirzaali M.J., Apachitei I., Zhou J., Zadpoor A.A. (2020). Multi-material additive manufacturing technologies for Ti-, Mg-, and Fe-based biomaterials for bone substitution. Acta Biomater..

[77] Putra N.E., Leeflang M.A., Taheri P., Fratila-Apachitei L.E., Mol J.M.C., Zhou J., Zadpoor A.A. (2021). Extrusion-based 3D printing of ex situ-alloyed highly biodegradable MRI-friendly porous iron-manganese scaffolds. Acta Biomater..

[78] Putra N.E., Leeflang M.A., Minneboo M., Taheri P., Fratila-Apachitei L.E., Mol J.M.C., Zhou J., Zadpoor A.A. (2021). Extrusion-based 3D printed biodegradable porous iron. Acta Biomater..

[79] Park J.W., Kang H.G. (2021). Application of 3-dimensional printing implants for bone tumors. Clin. Exp. Pediatr..

[80] Arjunan A., Robinson J., Baroutaji A., Tuñón-Molina A., Martí M., Serrano-Aroca Á. (2021). 3D Printed Cobalt-Chromium-Molybdenum Porous Superalloy with Superior Antiviral Activity. Int. J. Mol. Sci..

[81] Wang H., Su K., Su L., Liang P., Ji P., Wang C. (2019). Comparison of 3D-printed porous tantalum and titanium scaffolds on osteointegration and osteogenesis. Mater. Sci. Eng. C Mater. Biol. Appl..

[82] Rahimnejad M., Rezvaninejad R., Rezvaninejad R., França R. (2021). Biomaterials in bone and mineralized tissue engineering using 3D printing and bioprinting technologies. Biomed. Phys. Eng. Express.

[83] Li Z., Du T., Ruan C., Niu X. (2021). Bioinspired mineralized collagen scaffolds for bone tissue engineering. Bioact. Mater..

[84] Tonelli M., Faralli A., Ridi F., Bonini M. (2021). 3D printable magnesium-based cements towards the preparation of bioceramics. J. Colloid Interface Sci..

[85] Ma H., Feng C., Chang J., Wu C. (2018). 3D-printed bioceramic scaffolds: From bone tissue engineering to tumor therapy. Acta Biomater..

[86] Ma L., Wang X., Zhao N., Zhu Y., Qiu Z., Li Q., Zhou Y., Lin Z., Li X., Zeng X. (2018). Integrating 3D Printing and Biomimetic Mineralization for Personalized Enhanced Osteogenesis, Angiogenesis, and Osteointegration. ACS Appl. Mater. Interfaces.

[87] Owji N., Aldaadaa A., Cha J.R., Shakouri T., García-Gareta E., Kim H.W., Knowles J.C. (2020). Synthesis, Characterization, and 3D Printing of an Isosorbide-Based, Light-Curable, Degradable Polymer for Potential Application in Maxillofacial Reconstruction. ACS Biomater. Sci. Eng..

[88] Dewey M.J., Nosatov A.V., Subedi K., Shah R., Jakus A., Harley B.A.C. (2021). Inclusion of a 3D-printed Hyperelastic Bone mesh improves mechanical and osteogenic performance of a mineralized collagen scaffold. Acta Biomater..

[89] Nadi A., Khodaei M., Javdani M., Mirzaei S.A., Soleimannejad M., Tayebi L., Asadpour S. (2022). Fabrication of functional and nano-biocomposite scaffolds using strontium-doped bredigite nanoparticles/polycaprolactone/poly lactic acid via 3D printing for bone regeneration. Int. J. Biol. Macromol..

[90] Codrea C.I., Croitoru A.M., Baciu C.C., Melinescu A., Ficai D., Fruth V., Ficai A. (2021). Advances in Osteoporotic Bone Tissue Engineering. J. Clin. Med..

[91] Garot C., Bettega G., Picart C. (2021). Additive Manufacturing of Material Scaffolds for Bone Regeneration: Toward Application in the Clinics. Adv. Funct. Mater..

[92] Monfared M., Mawad D., Rnjak-Kovacina J., Stenzel M.H. (2021). 3D bioprinting of dual-crosslinked nanocellulose hydrogels for tissue engineering applications. J. Mater. Chem. B.

[93] Manzoor F., Golbang A., Jindal S., Dixon D., McIlhagger A., Harkin-Jones E., Crawford D., Mancuso E. (2021). 3D printed PEEK/HA composites for bone tissue engineering applications: Effect of material formulation on mechanical performance and bioactive potential. J. Mech. Behav. Biomed. Mater..

[94] Yu J., Xu Y., Li S., Seifert G.V., Becker M.L. (2017). Three-Dimensional Printing of Nano Hydroxyapatite/Poly(ester urea) Composite Scaffolds with Enhanced Bioactivity. Biomacromolecules.

[95] Dziaduszewska M., Wekwejt M., Bartmański M., Pałubicka A., Gajowiec G., Seramak T., Osyczka A.M., Zieliński A. (2019). The Effect of Surface Modification of Ti13Zr13Nb Alloy on Adhesion of Antibiotic and Nanosilver-Loaded Bone Cement Coatings Dedicated for Application as Spacers. Materials.

[96] Limongi T., Susa F., Allione M., di Fabrizio E. (2020). Drug Delivery Applications of Three-Dimensional Printed (3DP) Mesoporous Scaffolds. Pharmaceutics.

[97] Gu Z., Fu J., Lin H., He Y. (2020). Development of 3D bioprinting: From printing methods to biomedical applications. Asian J. Pharm. Sci..

[98] Bilici Ç., Tatar A.G., Şentürk E., Dikyol C., Koç B. (2022). Bisulfite-initiated crosslinking of gelatin methacryloyl hydrogels for embedded 3D bioprinting. Biofabrication.

[99] Melo P., Montalbano G., Fiorilli S., Vitale-Brovarone C. (2021). 3D Printing in Alginic Acid Bath of In-Situ Crosslinked Collagen Composite Scaffolds. Materials.

[100] Kuźmińska M., Pereira B.C., Habashy R., Peak M., Isreb M., Gough T.D., Isreb A., Alhnan M.A. (2021). Solvent-free temperature-facilitated direct extrusion 3D printing for pharmaceuticals. Int. J. Pharm..

[101] Dadras-Toussi O., Khorrami M., Abidian M.R. (2021). Femtosecond Laser 3D-printing of Conductive Microelectronics for Potential Biomedical Applications. Annu. Int. Conf. IEEE Eng. Med. Biol. Soc..

[102] Trikalitis V.D., Kroese N.J.J., Kaya M., Cofiño-Fabres C., Ten Den S., Khalil I., Misra S., Koopman B., Passier R., Scwach V. (2022). Embedded 3D printing of dilute particle suspensions into dense complex tissue fibers using shear thinning xanthan baths. Biofabrication.

[103] Shams E., Barzad M.S., Mohamadnia S., Tavakoli O., Mehrdadfar A. (2022). A review on alginate-based bioinks, combination with other natural biomaterials and characteristics. J. Biomater. Appl..

[104] Clark C., Yoo K.M., Sivakumar H., Strumpf K., Laxton A., Tatter S.B., Strowd R.E., Skardal A. (2022). Immersion bioprinting of hyaluronan and collagen bioink-supported 3D patient-derived brain tumor organoids. Biomed. Mater..

[105] Zhang Y., Chen H., Li J. (2022). Recent advances on gelatin methacrylate hydrogels with controlled microstructures for tissue engineering. Int. J. Biol. Macromol..

[106] Paxton N., Smolan W., Böck T., Melchels F., Groll J., Jungst T. (2017). Proposal to assess printability of bioinks for extrusion-based bioprinting and evaluation of rheological properties governing bioprintability. Biofabrication.

[107] Miyajima H., Kojima K., Touji H., Onodera K., Mukai M., Maruo S., Iijima K. (2024). Microfabrication of Gelatin Methacrylate/Hydroxyapatite Composites by Utilizing Alternate Soaking Process. ACS Biomater. Sci. Eng..

[108] Anjum S., Rahman F., Pandey P., Arya D.K., Alam M., Rajinikanth P.S., Ao Q. (2022). Electrospun Biomimetic Nanofibrous Scaffolds: A Promising Prospect for Bone Tissue Engineering and Regenerative Medicine. Int. J. Mol. Sci..

[109] Zhao T., Zhang J., Gao X., Yuan D., Gu Z., Xu Y. (2022). Electrospun nanofibers for bone regeneration: From biomimetic composition, structure to function. J. Mater. Chem. B.

[110] Liu K.D., Zhang D.J., Wang W. (2021). Nanoparticle-Based Drug Delivery System-A Target Strategy for Osteoarthritis Treatment. J. Nanomater..

[111] Gholami Farashah M.S., Javadi M., Mohammadi A., Soleimani Rad J., Shakouri S.K., Roshangar L. (2022). Bone marrow mesenchymal stem cell’s exosomes as key nanoparticles in osteogenesis and bone regeneration: Specific capacity based on cell type. Mol. Biol. Rep..

[112] Wang D., Cao H., Hua W., Gao L., Yuan Y., Zhou X., Zeng Z. (2022). Mesenchymal Stem Cell-Derived Extracellular Vesicles for Bone Defect Repair. Membranes.

[113] Bhujel B., Shin H.E., Choi D.J., Han I. (2022). Mesenchymal Stem Cell-Derived Exosomes and Intervertebral Disc Regeneration: Review. Int. J. Mol. Sci..

[114] Zha Y., Li Y., Lin T., Chen J., Zhang S., Wang J. (2021). Progenitor cell-derived exosomes endowed with VEGF plasmids enhance osteogenic induction and vascular remodeling in large segmental bone defects. Theranostics.

[115] Kang M., Lee C.S., Lee M. (2021). Bioactive Scaffolds Integrated with Liposomal or Extracellular Vesicles for Bone Regeneration. Bioengineering.

[116] Cheng R., Liu L., Xiang Y., Lu Y., Deng L., Zhang H., Santos H.A., Cui W. (2020). Advanced liposome-loaded scaffolds for therapeutic and tissue engineering applications. Biomaterials.

[117] Li Y., Bai Y., Pan J., Wang H., Li H., Xu X., Fu X., Shi R., Luo Z., Li Y. (2019). A hybrid 3D-printed aspirin-laden liposome composite scaffold for bone tissue engineering. J. Mater. Chem. B.

[118] Cisneros K., Chowdhury N., Coleman E., Ferdous T., Su H., Jennings J.A., Bumgardner J.D., Fujiwara T. (2021). Long-Term Controlled Release of Simvastatin from Photoprinted Triple-Networked Hydrogels Composed of Modified Chitosan and PLA-PEG Micelles. Macromol. Biosci..

[119] Wang W., Xiong Y., Zhao R., Li X., Jia W. (2022). A novel hierarchical biofunctionalized 3D-printed porous Ti6Al4V scaffold with enhanced osteoporotic osseointegration through osteoimmunomodulation. J. Nanobiotechnol..

[120] Chauhan A.S. (2018). Dendrimers for Drug Delivery. Molecules.

[121] Doosti-Telgerd M., Mahdavi F.S., Moradikhah F., Porgham Daryasari M., Bayrami Atashgah R., Dolatyar B., Akbari Javar H., Seyedjafari E., Shabani I., Arefian E. (2020). Nanofibrous Scaffolds Containing Hydroxyapatite and Microfluidic-Prepared Polyamidoamin/BMP-2 Plasmid Dendriplexes for Bone Tissue Engineering Applications. Int. J. Nanomed..

[122] Bose S., Sarkar N., Majumdar U. (2023). Micelle encapsulated curcumin and piperine-laden 3D printed calcium phosphate scaffolds enhance in vitro biological properties. Colloids Surf. B Biointerfaces.

[123] Mahmoudi M., Dargahi M., Mashayekhan S., Jahanmardi R., Baniasadi H. (2025). Structure-property-function relationships in 3D-printed gelatin/cellulose nanocrystals/n-HAP scaffolds for bone tissue repair. Int. J. Biol. Macromol..

[124] Salem N.A., ElShebiney S.A., Mabrouk M., Kishta M.S., Galal A.F., Osama L., Beherei H.H. (2025). Enhanced bone regeneration using mesenchymal stem cell-loaded 3D-printed alginate-calcium Titanate scaffolds: A Calvarial defect model study. Int. J. Biol. Macromol..

[125] Dutta S.D., Hexiu J., Moniruzzaman M., Patil T.V., Acharya R., Kim J.S., Lim K.T. (2025). Tailoring osteoimmunity and hemostasis using 3D-Printed nano-photocatalytic bactericidal scaffold for augmented bone regeneration. Biomaterials.

[126] Pahlevanzadeh F., Emadi R., Kharaziha M., Poursamar S.A., Nejatidanesh F., Emadi H., Aslani R., Moroni L., Setayeshmehr M. (2024). Amorphous magnesium phosphate-graphene oxide nano particles laden 3D-printed chitosan scaffolds with enhanced osteogenic potential and antibacterial properties. Biomater. Adv..

[127] Li S., Li Z., Yang J., Ha Y., Zhou X., He C. (2022). Inhibition of Sympathetic Activation by Delivering Calcium Channel Blockers from a 3D Printed Scaffold to Promote Bone Defect Repair. Adv. Healthc. Mater..

[128] Ma H., Yang C., Ma Z., Wei X., Younis M.R., Wang H., Li W., Wang Z., Wang W., Luo Y. (2022). Multiscale Hierarchical Architecture-Based Bioactive Scaffolds for Versatile Tissue Engineering. Adv. Healthc. Mater..

[129] Im S., Choe G., Seok J.M., Yeo S.J., Lee J.H., Kim W.D., Lee J.Y., Park S.A. (2022). An osteogenic bioink composed of alginate, cellulose nanofibrils, and polydopamine nanoparticles for 3D bioprinting and bone tissue engineering. Int. J. Biol. Macromol..

[130] Liu C., Qin W., Wang Y., Ma J., Liu J., Wu S., Zhao H. (2021). 3D Printed Gelatin/Sodium Alginate Hydrogel Scaffolds Doped with Nano-Attapulgite for Bone Tissue Repair. Int. J. Nanomed..

[131] Yin J., Mao X., Shang P., Chen S., Yang G., He H., He C., Zhou X. (2026). Synergistic 3D-bioprinted scaffold with multi-level adaptability for vascularized bone regeneration via osteogenesis-angiogenesis coupling. Mater. Today Bio.

[132] Gao Z., Song Z., Guo R., Zhang M., Wu J., Pan M., Du Q., He Y., Wang X., Gao L. (2024). Mn Single-Atom Nanozyme Functionalized 3D-Printed Bioceramic Scaffolds for Enhanced Antibacterial Activity and Bone Regeneration. Adv. Healthc. Mater..

[133] Liu L., Wu J., Lv S., Xu D., Li S., Hou W., Wang C., Yu D. (2023). Synergistic effect of hierarchical topographic structure on 3D-printed Titanium scaffold for enhanced coupling of osteogenesis and angiogenesis. Mater. Today Bio.

[134] Liu Y.Y., Dobricic M., Intini C., O’Brien F.J., Llorca J., Echeverry-Rendon M. (2026). Interface-engineered 3D-printed PCEC/collagen composite scaffold for large bone defect repair under static and mechanical stimulation. Colloids Surf. B Biointerfaces.

[135] Chen J., Qian Y., Li H., Zuo W., Sun W., Xing D., Zhou X. (2024). Lysophosphatidic Acid/Polydopamine-Modified nHA Composite Scaffolds for Enhanced Osteogenesis via Upregulating the Wnt/Beta-Catenin Pathway. ACS Appl. Mater. Interfaces.

[136] Kontogianni G.I., Bonatti A.F., De Maria C., Naseem R., Melo P., Coelho C., Vozzi G., Dalgarno K., Quadros P., Vitale-Brovarone C. (2023). Promotion of In Vitro Osteogenic Activity by Melt Extrusion-Based PLLA/PCL/PHBV Scaffolds Enriched with Nano-Hydroxyapatite and Strontium Substituted Nano-Hydroxyapatite. Polymers.

[137] Miao Y., Chen Y., Luo J., Liu X., Yang Q., Shi X., Wang Y. (2023). Black phosphorus nanosheets-enabled DNA hydrogel integrating 3D-printed scaffold for promoting vascularized bone regeneration. Bioact. Mater..

[138] Zhang X., He J., Qiao L., Wang Z., Zheng Q., Xiong C., Yang H., Li K., Lu C., Li S. (2022). 3D printed PCLA scaffold with nano-hydroxyapatite coating doped green tea EGCG promotes bone growth and inhibits multidrug-resistant bacteria colonization. Cell Prolif..

[139] Kim H.S., Lee J.H., Mandakhbayar N., Jin G.Z., Kim S.J., Yoon J.Y., Jo S.B., Park J.H., Singh R.K., Jang J.H. (2021). Therapeutic tissue regenerative nanohybrids self-assembled from bioactive inorganic core/chitosan shell nanounits. Biomaterials.

[140] Lv Z., Peng B., Ye Y., Xu H., Cai X., Liu J., Dai J., Bian Y., Wen P., Weng X. (2025). Bolstered bone regeneration by multiscale customized magnesium scaffolds with hierarchical structures and tempered degradation. Bioact. Mater..

[141] Putri N.R.E., Chen H., Kawazoe N., Rose F., Wildman R.D., Chen G. (2025). Tailoring cell behaviour by surface micropatterning and interconnected porous structure of gelatin/nano-silica/PLGA 3D composite scaffold for bone tissue engineering. RSC Adv..

